# Leveraging Enhanced Hi‐C Capture Analysis (eHiCA) to identify susceptibility genes in ambiguous GWAS loci for Alzheimer's disease: evidence for known and unusual suspects

**DOI:** 10.1002/alz70855_105108

**Published:** 2025-12-24

**Authors:** Karen Nuytemans, Liyong Wang, Luciana Bertholim Nasciben, Wanying Xu, Xiaoxiao Liu, Farid Rajabli, Katrina Celis, Leina Lu, Marla Gearing, David A. A. Bennett, Margaret E Flanagan, Sandra Weintraub, Changiz Geula, Theresa Schuck, William K. Scott, Derek M. Dykxhoorn, Anthony J Griswold, Margaret Pericak‐Vance, Juan I Young, Fulai Jin, Jeffery M Vance

**Affiliations:** ^1^ Dr. John T. Macdonald Foundation Department of Human Genetics, University of Miami Miller School of Medicine, Miami, FL, USA; ^2^ University of Miami Miller School of Medicine, Miami, FL, USA; ^3^ John P. Hussman Institute for Human Genomics, University of Miami Miller School of Medicine, Miami, FL, USA; ^4^ Department of Genetics and Genome Sciences, School of Medicine, Case Western Reserve University, Cleveland, OH, USA; ^5^ Department of Genetics and Genome Sciences, Case Western Reserve University, Cleveland, OH, USA; ^6^ Goizueta Alzheimer's Disease Research Center, Emory University, Atlanta, GA, USA; ^7^ Department of Neurological Sciences, Rush University Medical Center, Chicago, IL, USA; ^8^ Northwestern ADC Neuropathology Core, Northwestern University Feinberg School of Medicine, Chicago, IL, USA; ^9^ Department of Pathology and Laboratory Medicine, Institute on Aging and Center for Neurodegenerative Disease Research, The Perelman School of Medicine at the University of Pennsylvania, Philadelphia, PA, USA; ^10^ Department of Computer and Data Sciences, Case Western Reserve University, Celveland, OH, USA

## Abstract

**Background:**

The identification of susceptibility genes in noncoding genome‐wide association study (GWAS) loci remains a challenge as interactions between regulatory elements and targets can span several Mb and exceed 1:1 ratio of element‐to‐target. For several noncoding AD GWAS loci, the variant(s) and gene target(s) likely to be driving the association have been difficult to determine, with multiple or no clear candidate genes. To identify underlying susceptibility gene(s) in these “ambiguous” loci, we queried an in‐house high‐resolution Hi‐C database of genomic interactions in multiple genomic ancestries.

**Method:**

Hi‐C libraries were constructed using brain frontal cortex and iPSC‐derived AD relevant cells from donors matched for age, sex, and APOE genotype. We used Enhanced Hi‐C Capture analyses (eHiCA), incorporating the DeepLoop pipeline with 5kb resolution and 5kb bait windows surrounding the top GWAS and LD variants, to map chromatin loops using Hi‐C data. In our initial analyses, we applied eHiCA to uncover the regulatory architecture in six ambiguous loci from the Bellenguez et al GWAS in non‐Hispanic whites (NHW).

**Result:**

In the PICALM/EED locus, we found PICALM as the susceptibility gene with the strongest evidence, though lower‐level interaction with the EED promoter and regions surrounding GWAS LD variants were also observed. Interestingly, in the USP6NL/ECHDC3 and BCKDK/KAT8 loci, multiple candidate genes other than the closest genes to the LD variants were looped to the GWAS LD variants, including CELF2 and SETD1A, respectively. For two other loci (EPDR1/NME8 and MINDY2/ADAM10), the most likely candidates were the gene after which each locus was named (NME8 and ADAM10), with evidence supporting GWAS variants located in promoter or enhancer sites looping back to those genes. Interactions observed in the UNC5CL/TREM2 locus did not identify TREM2 or any other gene promoter as a likely target but showed interaction with an enhancer supporting potential indirect effects.

**Conclusion:**

This initial application of eHiCA on ambiguous AD GWAS loci demonstrates the utility of chromatin regulatory maps in nominating candidate genes of GWAS hits in non‐coding loci which lack clear candidates. This extended application of eHiCA will broaden our understanding of AD genomics overall.

